# Insider trading

**DOI:** 10.1038/s41597-023-02147-6

**Published:** 2023-04-26

**Authors:** Attila Balogh

**Affiliations:** Department of Finance, University of Melbourne, Melbourne, 3010 Australia

**Keywords:** Business, Economics

## Abstract

This paper describes a dataset capturing insider trading activity at publicly traded companies. Investors and investment analysts demand this information because executives, directors and large shareholders are expected to have more intimate knowledge of their company’s prospects than outsiders. Insider stock sales and purchases may reveal information about the firm’s business not disclosed in financial statements. They may also convey new information predictive of stock price movements if insiders can better interpret public information about the firm. Since mid-2003, the Securities and Exchange Commission has made these insider trading reports available to the public in a structured format; however, most academic papers use proprietary commercial databases instead of regulatory filings directly. This makes replication challenging as the data manipulation and aggregation processes are opaque and historical records could be altered by the database provider over time. To overcome these limitations, the presented dataset is created from original regulatory filings; it is updated daily and includes all information reported by insiders without alteration.

## Background & Summary

It is illegal for executives, directors, and blockholders to buy or sell stock in their firm based on proprietary information not generally available to the investing public. Nevertheless, identifying and successfully prosecuting insider trading is challenging due to the subtle distinctions between what is and isn’t legal^1–3^. For instance, studies by financial economists found that trading by target firm insiders during mergers and acquisitions can signal acquisition outcomes, suggesting that insiders know something about the pending deal that the investing public does not^4^. Research also shows that brokers of insider traders have an information advantage, finding that affiliated analysts issue more accurate earnings forecasts^5^. Moreover, a decision *not* to trade can also be informative. Research shows that future stock returns are lower following insider silence compared to insider selling, especially for firms with a higher litigation risk^6^.

However, while previous studies illustrate the effects of insider trading activity, they are often based on proprietary commercial databases. For example, the most common source of insider trading information is the Refinitiv (formerly Thomson Reuters) Insider Filings product. Commercially available databases are widely used in academic research, but they may be subject to opaque aggregation processes or data manipulation over time. These practices limit the ability of future researchers to replicate studies or guarantee the accuracy of their results. To help mitigate this ongoing issue, this paper presents the Layline Insider Trading dataset, a transparent and publicly available database capturing insider trading activity at publicly traded firms. Project Layline compiles data directly from the United States Securities and Exchange Commission (SEC) and is straightforward to employ in empirical analysis. The use of this unaltered, continuously updated data lowers barriers to entry in this important field and encourages new research that builds on the extensive prior work^7–76^ summarized in recent surveys^77,78^.

Publicly-traded companies and certain individuals in the US are legally required to file trading reports with the SEC. Section 16 of the Securities Exchange Act of 1934, in particular, requires that insiders report all changes in ownership by the end of the second business day following the transaction date. This requirement applies to officers in a policy-making function and their immediate family members; the firm’s directors; blockholders that are beneficial owners of more than 10% of the firm’s equity; and other persons affiliated with the insider.

Original insider trading records are submitted in Extensible Markup Language (XML) format to the SEC’s Electronic Data Gathering, Analysis, and Retrieval (EDGAR) system, which facilitates the creation of relational databases from filings. Reporting in XML format commenced on June 30, 2003, whereas prior transactions were filed in a plain text format. The Layline Insider Trading dataset acquires this information directly from EDGAR by downloading all individual filings and filing metadata, and parsing their XML content. This process offers additional benefits over commercial datasets by yielding a richer data set, facilitating replication by not altering observations, and providing direct links to the original sources.

Layline Insider Trading is one of the first datasets released under Project Layline, a research initiative that leverages high performance computing and data science tools to create publicly accessible datasets for research in financial economics. The project advances inclusivity by democratizing access to data and brings increased transparency to the field by promoting open science^79^. This initiative also includes datasets on the ownership structure of public firms;^80,81^ regulatory filings by activist hedge funds and other blockholders;^82,83^ and corporate filing metadata^84,85^. The Layline Insider Trading dataset is updated daily with all previous versions retained to encourage academic exploration of relevant and time-sensitive research questions. All datasets are hosted by Harvard Dataverse and they are also regularly updated on Kaggle^86^.

## Methods

The Python code developed for this project has two main components: acquisition and processing. The acquisition script downloads the quarterly Master Index of EDGAR Dissemination Feed files for each year and quarter starting from 2003, ending with the most recent one^87^. It identifies all Form 3, 3/A, 4, 4/A, 5, and 5/A filings in the master index and downloads both the metadata and the full filing to a local directory structure. Downloads follow a naming convention based on the form type, the filer’s Central Index Key (CIK), and each filing’s unique identifier, i.e., its Accession Number. The processing scripts parse the elements in the XML file and save them to a comma-separated values (CSV) file.

As of early 2023, the raw data depository contained over 18 million files. Because the EDGAR system limits the download rate to no more than ten items per second, downloading all filings can take over a month^88^. The acquisition script creates and updates a Structured Query Language (SQL) database using SQLite to track successfully downloaded filings. Running the script multiple times ensures that attempts are made to download filings missed during prior executions, either because the EDGAR service was temporarily unavailable or due to a 403 Forbidden hypertext transfer protocol (HTTP) standard response code in instances when the script inadvertently exceeds the download limit. The acquisition script incorporates rate limiting using the *ratelimit* Python package. However, the limit may be exceeded if multiple acquisition scripts run simultaneously on the same network or have the same user agent in request headers embedded in the Python code.

The processing scripts create seven individual CSV files, as shown in Table 1. The variable names in the CSV files follow the naming convention of the XML tags in the original filings^89^. The *Submission* table includes the filing’s metadata and header information; details of the filing entities are stored in the *Reporting owners* table; *Non-derivative* and *Derivative* transactions are divided into respective tables; as are *Footnotes* and *Signatures*. A separate header table is provided that includes all filing metadata, even for reports that are not submitted in XML format. Each CSV dataset is accompanied by an error log listing filings that include non-XML compatible strings or were unavailable to download and returned a 404 HTTP standard response code.

**Table 1:** Layline insider trading dataverse files.

File name	Content
lit_panel.zip	Final merged dataset
lit_submission.zip	Submission table
lit_reportingowner.zip	Reporting owners table
lit_nonderiv.zip	Non-derivative securities
lit_deriv.zip	Derivative securities
lit_footnotes.zip	Footnotes table
lit_signatures.zip	Signatures table
lit_header.zip	Header metadata
panel_dataset.do	Stata code for merging tables
panel_dataset.py	Python code for merging tables

This table provides the list of files made available in the repository and their brief description.

## Data Records

The Layline Insider Trading dataset is available in the Layline Dataverse repository (https://dataverse.harvard.edu/dataverse/layline) and includes three main types of regulatory filings pertaining to changes of firm ownership^90^. Form 3 filings are submitted when a person first becomes an insider; these initial disclosures must be filed within ten calendar days. Form 4 filings must be filed when an insider executes a transaction in the company’s securities, such as purchasing or selling shares or trading in derivative instruments. These forms need to be filed within two business days following a transaction. Form 5 filings are annual insider trading reports due within 45 days of the company’s fiscal year-end. They include transactions not required on Form 4 filings, such as smaller transactions that do not exceed $10,000 in a six-month period. In the case of mistakes, all three types of filings can be corrected with an amendment, denoted as Form 3/A, 4/A, or 5/A filings.

Each dated version of the structured dataset includes six CSV files, which are organized into Submission, Reporting owners, Non-derivative securities, Derivative securities, Footnotes, and Signatures tables. The tables are stored separately because of the many-to-many relationship between them, and they can be merged on the unique Accession Number identifier to create customized representations of the data. Table 2 lists all variable names in each table, and Table 3 provides an overview of the filings with the annual breakdown of the sample. The majority of the filings in the dataset are Form 4 filings or their amendments. Since the XML reporting requirement came into effect on June 30, 2003, the first year is expected to have half as many observations, not considering annual and seasonal trends.

**Table 2:** Variable names.

Submissions	Reporting owners	Non-derivative transactions	Derivative transactions
periodOfReport	Footnotes	Signatures	
URL	URL	URL	URL
acceptanceDatetime	accessionNumber	accessionNumber	accessionNumber
accessionNumber	filingDate	filingDate	filingDate
filerCik	filerCik	filerCik	filerCik
type	rptOwnerCik	transactionType	transactionType
publicDocumentCount	rptOwnerName	tableRow	tableRow
period	rptOwnerStreet1	securityTitle	securityTitle
filingDate	rptOwnerStreet2	securityTitleFn	securityTitleFn
dateOfFilingDateChange	rptOwnerCity	transactionDate	conversionOrExercisePrice
ownerName	rptOwnerState	transactionDateFn	conversionOrExercisePriceFn
ownerCik	rptOwnerZipCode	deemedExecutionDate	transactionDate
ownerSic	rptOwnerStateDescription	deemedExecutionDateFn	transactionDateFn
ownerStateOfIncorporation	isDirector	transactionFormType	deemedExecutionDate
ownerFiscalYearEnd	isOfficer	transactionCode	deemedExecutionDateFn
ownerFormType	isTenPercentOwner	equitySwapInvolved	transactionFormType
ownerAct	isOther	transactionCodeFn	transactionCode
ownerFileNumber	officerTitle	transactionTimeliness	equitySwapInvolved
ownerFilmNumber	otherText	transactionTimelinessFn	transactionCodeFn
ownerBusinessStreet1		transactionShares	transactionTimeliness
ownerBusinessStreet2		transactionSharesFn	transactionTimelinessFn
ownerBusinessCity		transactionPricePerShare	transactionShares
ownerBusinessState		transactionPricePerShareFn	transactionSharesFn
ownerBusinessZip		transactionAcquiredDisposedCode	transactionTotalValue
ownerBusinessPhone		transactionAcquiredDisposedCdFn	transactionTotalValueFn
ownerMailingStreet1		sharesOwnedFollowingTransaction	transactionPricePerShare
ownerMailingStreet2		sharesOwnedFolwngTransactionFn	transactionPricePerShareFn
ownerMailingCity		valueOwnedFollowingTransaction	transactionAcquiredDisposedCode
ownerMailingState		valueOwnedFolwngTransactionFn	transactionAcquiredDisposedCdFn
ownerMailingZip		directOrIndirectOwnership	exerciseDate
issuerConformedName		directOrIndirectOwnershipFn	exerciseDateFn
issuerCIK		natureOfOwnership	expirationDate
issuerSic		natureOfOwnershipFn	expirationDateFn
issuerIRSNumber			underlyingSecurityTitle
issuerStateOfIncorporation			underlyingSecurityTitleFn
issuerFiscalYearend			underlyingSecurityShares
issuerBusinessStreet1			underlyingSecuritySharesFn
issuerBusinessStreet2			underlyingSecurityValue
issuerBusinessCity			underlyingSecurityValueFn
issuerBusinessState			sharesOwnedFollowingTransaction
issuerBusinessZip			sharesOwnedFolwngTransactionFn
issuerBusinessPhone			valueOwnedFollowingTransaction
issuerMailingStreet1			valueOwnedFolwngTransactionFn
issuerMailingStreet2			directOrIndirectOwnership
issuerMailingCity			directOrIndirectOwnershipFn
issuerMailingState			natureOfOwnership
issuerMailingZip			natureOfOwnershipFn
issuerFormerName			
issuerFormerDate			
schemaVersion			
documentType			
dateOfOriginalSubmission	URL	URL	
notSubjectToSection 16	accessionNumber	accessionNumber	
issuerCik	filingDate	filingDate	
issuerName	filerCik	filerCik	
issuerTradingSymbol	id	signatureName	
remarks	content	signatureDate	

This table provides the list of variable names in each table of the dataset extracted from Form 3, Form 4 and Form 5 filings submitted to the SEC’s EDGAR system. The Submissions table mainly captures filing metadata, except for the variables starting with schemaVersion that are extracted from the body of the structured filing and may duplicate metadata fields, such as the Issuer’s CIK identifier.

**Table 3:** Annual breakdown of insider trading filings.

	Form 3	3/A	Form 4	4/A	Form 5	5/A	Total
2003	11,541	717	125,684	5,877	2,103	165	146,087
2004	22,871	1,582	232,278	10,438	9,904	652	277,725
2005	22,679	1,616	230,344	9,065	8,269	386	272,359
2006	21,903	1,396	227,994	9,349	7,485	348	268,475
2007	24,845	1,348	234,776	8,725	6,461	294	276,449
2008	17,565	1,186	217,751	7,696	5,686	214	250,098
2009	14,286	1,277	185,988	5,989	5,235	210	212,985
2010	15,403	828	194,888	5,629	4,631	156	221,535
2011	14,900	749	190,117	5,349	4,453	120	215,688
2012	14,020	709	192,604	5,095	4,167	164	216,759
2013	14,648	712	193,175	4,799	3,987	136	217,457
2014	16,582	791	193,757	4,835	4,022	124	220,111
2015	15,174	734	191,907	5,026	3,451	105	216,397
2016	13,749	693	182,192	4,389	3,226	73	204,322
2017	14,010	649	180,801	3,891	3,079	66	202,496
2018	14,196	601	180,460	3,673	3,040	81	202,051
2019	14,034	487	175,430	3,748	2,591	44	196,334
2020	16,022	581	180,626	3,695	2,590	56	203,570
2021	23,128	788	194,453	3,672	2,390	43	224,474
2022	14,018	568	182,301	3,455	2,482	80	202,904
Total	335,574	18,012	3,887,526	114,395	89,252	3,517	4,448,276

This table presents the annual breakdown of statements of beneficial ownership of securities filed in XML format by insiders and obtained from the SEC’s EDGAR system. Form 3 filings are the initial statements, Form 4 filings are the statements of changes, and Form 5 are the annual statements. The sample period starts on May 5, 2003 and ends on December 31, 2022.

### Submission metadata and header

The Submission table contains information on the two transacting parties: the issuer of the securities (the company) and the reporting entity, who is the company insider and either a person, partnership, or company. The EDGAR system stores each individual filing under both the issuer’s and the reporting entity’s accounts. As a result, all transaction records will have at least two observations that are identical for all variables except for the filing’s Uniform Resource Locator (URL). Additionally, some insider trading reports are filed by multiple reporting entities. For example, one insider may be a company that is a blockholder in the issuer, and the blockholder’s employee may serve as a director on the issuer’s board. A filing with two reporting owners has three observations in the Submission dataset relating to the same report: one by the issuer and one each by the two reporting owners. One example is the filing with Accession Number 0001567619-22-018898 in the Header table^91^.

Each report is identified by its SEC Accession Number, whereas issuers and reporting entities are identified by their Central Index Key. Removing duplicates in the Header table by keeping all unique Accession Number–Issuer CIK combinations yields a unique record of each insider trading filing. Stata and Python examples for this data-cleaning step are provided in the data repository.

**Fig. 1:**
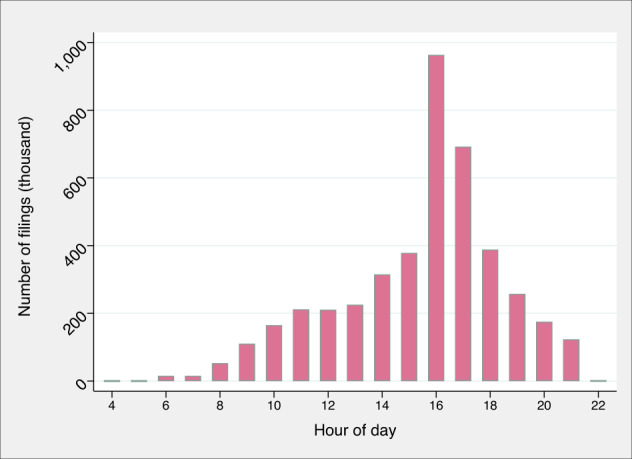
Number of filings per hour of the day.

The presented dataset is the first to include the date and time the SEC accepted the filing. Figure 1 provides insight into the heterogeneity of filing times across the sample. This variable offers new research opportunities in accounting and finance research to explore whether some insiders strategically time their disclosure to certain days of the week or hours of the day, such as after trading hours. Using this more precise measure, future researchers may build on prior work and study the timeliness of reporting by examining whether some insiders are more likely to delay filing a report after a transaction^92,93^.

### Reporting owners

The Reporting owners table includes information on the reporting entity for the insider trading transaction, comprising their CIK identifier, name, address, and their filing capacity (i.e., officer, director, blockholder, or other). If the reporting entity is an officer of the firm or other insider, the filing also contains the nature of the relationship in the officerTitle and otherText fields. On occasion, the officerTitle field takes the value of “See remarks”, in which case, the remark field includes this information. Since only one remark field is allowed for each filing, this field is stored in the Submission table to avoid duplication. Researchers are encouraged to develop methods to classify some of the more common values in these columns and share them publicly in order to promote replication and consistency across studies.

Because each report is saved in EDGAR under both the issuer and the reporting owners, a filing with one insider will have two identical observations in this table. A filing with three reporting entities will have twelve observations because the issuer and each of the three reporting owners will list the nature of the insider relationship for the three insiders separately. The number of expected rows for each filing in the Reporting owners table is ((number of reporting owners + 1) × number of reporting owners).

### Non-derivative and derivative securities

The two key components of the dataset are Table I, which includes non-derivative securities, and Table II for derivative securities, stored in separate CSV files. Each row in these tables describes a transaction, including the title of the security, the transaction date, a transaction code, the number of securities, and the price. Alternatively, a row may also describe a holding report if the insider’s securities holding spans multiple categories.

The transaction code is a single letter denoting the nature of the transaction. The most common codes are “S” and “P”, which stand for open market or private sale and purchase of securities, respectively. As shown in Table 4, grants and awards are the second most common type of transaction, denoted by the transaction code “A”. More detailed descriptions of these transaction codes is available on the SEC’s web site^94^.

**Table 4:** Transaction codes.

Code	Description	Derivative	Non-derivative
A	Grant, award or other acquisition pursuant to Rule 16b-3(d)	1,323,789	1,126,818
C	Conversion of derivative security	87,784	76,840
D	Disposition to the issuer of issuer equity securities pursuant to Rule 16b-3(e)	174,437	139,944
E	Expiration of short derivative position	2,050	87
F	Payment of exercise price or tax liability in accordance with Rule 16b-3	6,788	666,226
G	Bona fide gift	12,456	203,948
H	Expiration or cancellation of long derivative position with value received	2,553	70
I	Discretionary transaction in accordance with Rule 16b-3(f)	5,068	16,160
J	Other acquisition or disposition	108,704	296,151
L	Small acquisition under Rule 16a-6	992	22,371
M	Exercise or conversion of derivative security exempted pursuant to Rule 16b-3	1,016,839	979,705
O	Exercise of out-of-the-money derivative security	666	562
P	Open market or private purchase of non-derivative or derivative security	40,810	904,917
S	Open market or private sale of non-derivative or derivative security	17,853	2,995,205
U	Disposition pursuant to a tender of shares in a change of control transaction	3,981	12,022
W	Acquisition or disposition by will or the laws of descent and distribution	359	3,202
X	Exercise of in-the-money or at-the-money derivative security	43,281	28,958
Z	Deposit into or withdrawal from voting trust	145	1,905
Total		2,848,555	7,475,091

This table provides a breakdown and description of transaction codes in the Layline Insider Trading dataset for the sample period in Table 3.

Not all filings will have an associated securities table. Exit filings, for example, are final reports noting that the insider is no longer subject to regular reporting, which is also indicated by the notSubjectToSection 16 variable taking the value of one^95^. Finally, two additional fields include the number of securities owned after the transaction and whether it is a direct or indirect holding.

### Footnotes

As shown in Table 2, nearly every variable in the Non-derivative and Derivative tables has an associated footnote field, denoted by the “Fn” suffix, such as securityTitleFn for the securityTitle variable. The EDGAR web site shows these fields towards the bottom of each filing under the “Explanation of Responses” heading, above the remarks and signature block sections. These footnote variables take the values of F*x*, where *x* is an integer if the associated variable has an associated footnote. They can be merged to the Non-derivative and Derivative tables using the filing’s Accession Number and footnote identifier.

### Signatures

The Signatures table includes the name of the person who signed the filing, sometimes with their job title or signing capacity, in the signatureName column and the date of signature in the signatureDate column^96^. As with the other tables, signatures can be merged to each filing or transaction using the Accession Number variable. While it can be justified to de-duplicate this table prior to merging it with other tables in the dataset, it is worth noting that this table can include genuine duplicates for filings submitted by multiple reporting entities but signed by the same person^97^. It is expected that for each unique issuer and reporting owner, there will be one fewer signature because only reporting owners sign the filing. However, there are exceptions to this guideline if one reporting owner provides multiple signatures for a single filing^98^.

## Technical Validation

This section introduces two methods to validate the presented dataset. The first involves running the acquisition and processing scripts on multiple systems on distinct networks and comparing the output datasets. Access to files on the EDGAR system may be intermittent, and the acquisition script may encounter 403 Forbidden or 404 Not Found HTTP standard response codes when attempting to download certain filings. It is also possible that filings become corrupted during download or at rest. Running the acquisition script multiple times minimizes these errors and ensures no 403 Forbidden response codes are returned and recorded in error logs. The processing scripts are then executed on each computer system and the output files are saved for reference. In untabulated analysis, I compared the four sets of datasets and found them to be the same. The base dataset and three additional validation datasets are available in the data repository with the Stata code that offers a method for comparing them^99^. Downloading regulatory filings from EDGAR at different points in time can yield differences in three variables. The SEC may allow the official filing date to be adjusted after submission, in which case the filingDate variable changes and the dateOfFilingDateChange variable is populated^100^. Finally, the issuer’s fiscal year-end is a header-type variable as opposed to a historical value. When it changes, the issuerFiscalYearend variable will change for all filings, including those submitted prior to the change. One of the three validation datasets was downloaded at an earlier point in time, and the comparison Stata code was adjusted to highlight changes in the three variables.

The second validation involves comparing the output datasets to the Refinitiv Insider Filings datasets obtained from Wharton Research Data Services (WRDS). The downloaded Refinitiv “Table 1 Stock Transactions” and “Table 2 Derivative Transactions” datasets were last updated on January 3, 2023 and cover a period ending on December 30, 2022. This matches the time period covered by the first published version of the Layline dataset presented here. In contrast to commercially available databases such as Refinitiv, the Layline Insider Trading dataset offers insider trading reports in their original and unaltered form. The following section will highlight some inconsistencies encountered in comparing the presented dataset to the Refinitiv Insider Filings database.

The SEC also makes insider trading filings available after the end of each quarter in a tab-separated values file^101^. This paper will not include an analysis of this data source because a cursory review reveals that it does not appear to be a comprehensive dataset.

### Data coverage

Table 5 compares the Layline and Refinitiv Insider Trading datasets with an annual breakdown between 2004 and 2022, the overlapping time period with full annual coverage. Reporting in XML format became mandatory from June 30, 2003, but the EDGAR system and the presented datasets include XML filings from May 5, 2003. The Insiders column tabulates the number of unique reporting owners and shows that the presented dataset includes nearly twenty percent more reporting owners than Refinitiv. This discrepancy is likely explained by Refinitiv only including one insider for each filing, even when multiple insiders jointly report transactions. These figures should also be interpreted in the context of Refinitiv’s cleansing indicator, and the common practice of excluding some 6.5 percent of observations the data provider considers invalid (cleanse indicators A, S, and W). Accordingly, academic research based on this most commonly used dataset will likely be limited to a sub-sample of 75 percent of reported transactions.

**Table 5:** Summary comparison with the Refinitiv Insider Trading dataset.

	Insiders		Issuers		Form 3		Form 4		Form 5	
	Layline	Refinitiv	Layline	Refinitiv	Layline	Refinitiv	Layline	Refinitiv	Layline	Refinitiv
2004	77,815	71,861	8,813	8,251	24,453	13,523	242,716	237,691	10,556	9,638
2005	77,640	71,097	8,802	8,206	24,295	12,056	239,409	233,794	8,655	7,778
2006	77,260	70,519	8,957	8,113	23,299	11,980	237,343	233,062	7,833	6,788
2007	77,982	70,763	8,973	8,342	26,193	12,812	243,501	237,292	6,755	5,993
2008	71,857	65,780	8,528	7,762	18,751	9,214	225,447	220,113	5,900	5,334
2009	64,951	59,140	7,724	7,188	15,563	7,657	191,977	188,534	5,445	5,054
2010	64,976	53,400	7,368	6,379	16,231	6,020	200,517	166,594	4,787	4,169
2011	63,438	55,843	7,157	6,490	15,649	7,062	195,466	184,613	4,573	3,911
2012	61,585	55,816	6,633	6,188	14,729	7,429	197,699	194,899	4,331	4,042
2013	61,510	55,385	6,536	6,039	15,360	7,793	197,974	194,032	4,123	3,844
2014	62,627	56,301	6,520	6,108	17,373	8,612	198,592	193,721	4,146	3,756
2015	61,912	54,522	6,597	6,127	15,908	7,969	196,933	183,205	3,556	3,004
2016	59,798	53,399	6,287	5,816	14,442	10,631	186,581	174,508	3,299	3,025
2017	59,529	52,320	6,027	5,684	14,659	12,328	184,692	165,172	3,145	2,604
2018	58,946	53,940	5,967	5,702	14,797	12,986	184,133	179,827	3,121	2,851
2019	57,720	53,349	5,717	5,518	14,521	12,990	179,178	174,663	2,635	2,476
2020	59,202	52,565	5,901	5,685	16,603	12,690	184,321	171,744	2,646	2,468
2021	67,209	59,371	6,620	6,367	23,916	19,388	198,125	190,411	2,433	2,220
2022	62,032	57,039	6,284	6,024	14,586	12,237	185,756	182,295	2,562	2,385
Total	267,151	229,475	20,226	18,310	341,328	205,377	3,870,360	3,706,170	90,501	81,340

This table compares the presented Layline Insider Trading dataset and the Refinitiv Insider Trading dataset between 2004 and 2022. The Insiders and Issuers columns show the number of unique entities. The columns for Forms 3, 4, and 5 represent the number of those filings each year, including amendments.

The Layline dataset also covers over 13 percent more issuers, but Refinitiv does not include CIK identifiers, which makes it challenging to identify which filings are missing. The presented dataset is more comprehensive across all filing types, as the annual breakdown shows in Table 5. In order to gain sufficient confidence that all XML reports filed in EDGAR are downloaded and processed, the acquisition and processing scripts are regularly executed on multiple systems, and the independently generated datasets are compared to ensure that the daily updates contain all insider trading reports. Future work is encouraged to replicate the data acquisition and generation steps to verify the accuracy of the final output.

### Insider classification

Over 16 percent of insider filings are submitted by multiple entities during the sample period, as shown in Table 6. An executive of the firm may file them with their spouse or family trust, or they may be filed by an affiliated group of investors. These filings can reveal important differences in motivation and opportunistic behavior^102^. An examination of the Refinitiv data shows that it only records the identity of the first reporting owner and discards all other reporting entities. Because the order of reporting entities seems arbitrary in each filing, this approach may create measurement error in empirical research.

**Table 6:** Multiple reporting entities.

	Filings	Percentage	Cumulative
Officer	1,780,456	40.03	40.03
Director	1,662,432	37.37	77.40
Blockholder	182,620	4.11	81.50
Other	95,609	2.15	83.65
Officer - Director	472,284	10.62	94.27
Officer - Blockholder	4,696	0.11	94.38
Officer - Other	13,246	0.30	94.67
Officer - Director - Blockholder	110,018	2.47	97.15
Officer - Director - Other	4,838	0.11	97.26
Officer - Blockholder - Other	212	0.00	97.26
Officer - Director - Blockholder - Other	3,418	0.08	97.34
Director - Blockholder	70,934	1.59	98.93
Director - Other	17,582	0.40	99.33
Director - Blockholder - Other	5,036	0.11	99.44
Blockholder - Other	24,895	0.56	100.00
Total	4,448,276	100.00	

This table lists the number of filings where reporting owners act in their sole capacity as officers, directors, blockholders, or other insiders, and the number of filings with multiple types of relationships between the reporting owner and the issuer for the sample period described in Table 3.

This shortcoming is illustrated by the July 8, 2004 filing of buyout investor Harold Simmons in Vahli Inc., jointly submitted by Simmons with Contran Corp^103^. Because Contran is listed as the first entity in the SEC filing, Refinitiv shows this trade was carried out by a “beneficial owner of more than 10% of a class of security” under its Document Control Number (DCN) 071171227. The entry disregards the fact that Simmons was Chairman of the Board at Valhi at the time, and he is classified both in the filing and in the firm’s annual report as a director as well as an executive officer. The original XML data provided in EDGAR and the presented dataset allow for the appropriate identification of these observations as transactions carried out by insiders that are executives, directors, and blockholders at the same time.

Business and mailing addresses, the state of incorporation, and industry classification records are also retained for additional reporting entities but discarded by Refinitiv. As an additional benefit, the presented dataset also facilitates the identification of corporate group structures, which may present new avenues for research^104^. Given the non-trivial incidence of joint filings by multiple entities, future research may also attempt to replicate prior work and examine whether this measurement error in the Refinitiv dataset yields new and different findings.

### Link to original filings

The presented dataset includes a URL for each SEC report, allowing a direct method for cross-referencing each observation with the regulatory filing as it was submitted to EDGAR. The Layline dataset also includes the original CIK identifier for both the reporting entity and the issuer, which are masked by Refinitiv and replaced by proprietary identifiers. These features are important as transactions reported in Refinitiv are challenging to trace back to the original filings.

### Data aggregation

An additional benefit of the presented dataset is that it includes original observations without the aggregation employed by commercial datasets. Aggregation practices that are opaque, arbitrary, or undocumented may mask subtle differences potentially important for empirical analysis. Illustrating this point is an example of a stock award by ArvinMeritor Inc. to Vice President and Controller Rakesh Sachdev. The original filing reports that after an award of 5,000 shares on January 2, 2004, Mr. Sachdev held 375 shares directly, 1,311 through an employee benefit trust, and 12,462 in restricted stock^105^. The latter two are listed as indirect stock ownership in ArvinMeritor Inc. In contrast, Refinitiv breaks out the indirect ownership of the 1,311 shares held via the employee benefit trust; however, it combines the other two observations and lists them as direct stockholding totaling 12,837 shares (see Refinitiv DCN 04500028).

Researchers may form different opinions as to whether restricted stock is a direct or indirect holding, as these shares have been awarded to the executive but are held by the company because they are not vested. The important point is that this aggregation is undocumented by Refinitiv, and it is difficult to verify whether it is applied consistently across observations. The presented dataset makes it feasible to examine whether restricted stock holdings by insiders influence corporate policies. It may be hypothesized, for instance, that restricted stock holdings lower risk-taking by executives and limit investment in research and development, which are ultimately empirical questions to explore.

### Footnotes and remarks

**Fig. 2:**
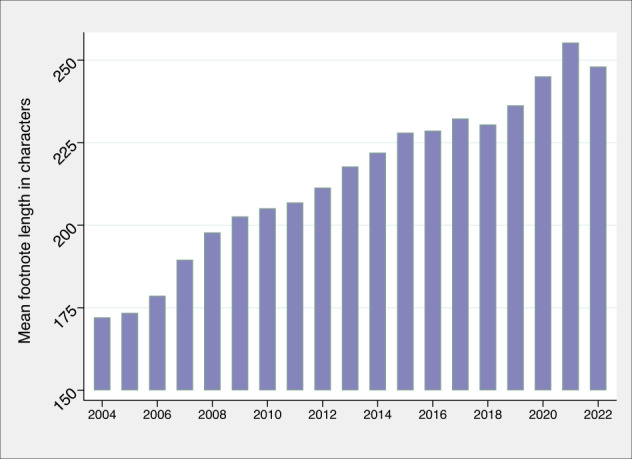
Mean footnote length per year.

The presented dataset is the first to include all explanatory footnotes to facilitate building on prior work exploring the relevance of these supplementary disclosures^106^. The potential significance of the Footnotes table is also highlighted by the increased use of this field, as shown in Fig. 2.

To illustrate the importance of footnotes, consider again the ArvinMeritor Inc. stock award to Rakesh Sachdev. The footnotes reveal that the 1,311 indirect holdings relate to “[s]hares purchased periodically and held in ArvinMeritor common stock funds in an employee benefit trust established under the ArvinMeritor, Inc. Savings Plan”^105^. Refinitiv groups these shares with the 12,462 that were “[h]eld by the issuer to implement restrictions on transfer unless and until certain conditions are met”^105^. Employing textual analysis on footnotes reveals new avenues of research that prior datasets could not accommodate.

In addition, the Layline dataset also includes the textual remarks field often used for longer job title descriptions not included in the dedicated officerTitle field. Access to job titles in both the officerTitle and remarks fields allows researchers to develop their own classification schemes instead of relying on the categories determined by commercial data providers. For example, Thomas J. Munson is listed as Chief Credit Officer and Executive Vice President in a March 2022 filing, whereas under Refinitiv’s DCN 21722797, he is listed simply as an Officer and EVP^107^. Future research may explore whether this richer and more granular classification reveals heterogeneity in trading patterns across executives. As previously described, the Securities Exchange Act of 1934 requires that officers in policy-making functions file insider trading reports, but it does not explicitly prescribe role titles. The presented dataset makes it feasible to identify job titles that firms consider policy-making functions and explore industry and temporal dynamics.

## Usage Notes

This section provides a case study example highlighting suggested data-cleaning steps and methods to collapse the data into a transaction-level panel for empirical analysis. The goal is to create a panel dataset offering insight into the prevalence of direct sale and purchase transactions by company insiders. The Stata code and its Python equivalent for merging CSV files and creating the summary tables included in this paper are available in the repository, along with the resulting panel dataset.

The first step in creating the panel involves loading the Submission table and removing the duplicates that occur because the same filing is recorded for the issuer and each reporting owner. As a general rule, this table should not contain duplicate observations that are the same across all fields. Filings are stored under both the issuer and the reporting owner, so they will have different filerCik values, but all other variables will be the same. A data cleaning step should keep observations with distinct Accession Number–Issuer CIK combinations, which yields a table without duplicates by only keeping one row per filing. At that stage, the filerCik variable should be removed from the dataset because it will randomly indicate either the issuer or the reporting entity.

Next, inspecting individual observations in the Reporting owners table reveals that approximately 16 percent of transactions are carried out by more than one reporting owner who can have various relationships with the firm. Filers are not always exclusively only an officer, director, blockholder, or other insider, as shown in Table 6. When the issuer is also a reporting owner, there is only one filer, which happens in a small number of filings. The first step in creating a transaction-level panel involves generating filing-level indicators on the relationship between the issuer and reporting owner before collapsing the Reporting owners table to the Accession Number level.

It is worth noting that the variable values denoting the relationship between the issuer and the reporting entity (i.e., isDirector, isOfficer, isTenPercentOwner, isOther) are not uniform across observations. In most cases they take on the values of either 0 or 1, but they can also be recorded as “false” or “true” for some filings. A cleaning step can turn these values into uniform dummy variables, as illustrated in the included Stata code.

The Non-derivative and Derivative securities tables (Tables I and II) include two main types of reports: transactions and holdings. The default place to list the holding balance after the reported transaction is column 5 of Table I and column 9 of Table II, along with the transaction. Often that completes the report, but when securities are held in the name of multiple entities, there may be multiple holding report lines for each transaction, with explanations provided in footnotes^108^.

Transactions in the Non-derivative and Derivative securities tables are expected to be duplicated: first under the issuer and then under the reporting owner. Filings that feature additional reporting entities will yield additional duplicates for each transaction. Nonetheless, removing seemingly identical observations from the Non-derivative and Derivative tables is inappropriate, as the reporting owner could have executed two identical transactions on the same day^109,110^. The proper method for removing duplicate observations is by creating a sequential table row identifier for each Accession Number and Filer CIK group, then removing observations with duplicate variables except for the Filer CIK. While it is not included in the original filings, this tableRow variable is created during the parsing process. In addition, the accompanying Stata code also offers a method to remove duplicates. It provides evidence that each observation (transaction or holding statement) in the Non-derivative and Derivative securities tables has exactly as many duplicates as the number of individual filers. This analysis directly verifies that no filings were inadvertently processed twice. It also points to instances with a single filer in cases where the issuer is also a reporting entity^111^.

One of the challenges in using this dataset for empirical research in finance is that the description of the traded security in the underlyingSecurityTitle field is provided in a non-standardized string form, such as “common stock”. Publicly traded securities are required to have a unique Committee on Uniform Securities Identification Procedures (CUSIP) identifier, but they are not included in the EDGAR filing. A ticker symbol is recorded in the Submission table, which is likely valid for the specific transaction date; however, trading symbols are not necessarily exclusive to the firm and may be reused by different firms over time. It also appears that there are no automated checks in EDGAR to verify the ticker symbol. The issuer’s CIK identifier is a verified field that researchers may use to link the insider trading report to commonly used stock price and financial statement data sources, such as the Center for Research in Security Prices (CRSP) datasets and Compustat.

A common error entails reporting the total monetary value of the transaction (i.e., number of securities times the price of a single security) instead of the price of a single security for the price variable. A potential data cleaning approach could take price values from the filings of the same issuer and trading symbol from adjacent periods, such as the preceding calendar month, and evaluate their median value against both the included price value and an imputed price, calculated as the listed price divided by the amount value. Researchers may also use other stock price datasets, such as CRSP, to evaluate misreporting in the price field and remove or change observations if they fall outside a pre-determined range. I encourage new contributions to the code and will share them with the dataset as additional encouragement to explore this exciting field in financial economics.

## Data Availability

The code used for the data normalization and merging steps was created and run in Stata/MP 17.0 and is made available in the data repository^90^. The acquisition and processing scripts are not shared publicly because EDGAR servers may block the simultaneous use of the same acquisition script based on the user agent in request headers. However, downloading regulatory filings via HTTP follows a standard procedure, and parsing XML files in Python using the lxml library is also well-documented.
